# Fatty Acid Enrichment of Corn Extrudates with Hemp Seeds

**DOI:** 10.3390/molecules30061390

**Published:** 2025-03-20

**Authors:** Marta Igual, David Gimeno, Purificación García-Segovia, Javier Martínez-Monzó, Juliana Navarro-Rocha

**Affiliations:** 1 i-Food Group, Instituto Universitario de Ingeniería de Alimentos-FoodUPV, Universitat Politècnica de València, Camino de Vera Vera s/n, 46021 Valencia, Spain; pugarse@tal.upv.es (P.G.-S.); xmartine@tal.upv.es (J.M.-M.); 2 Department of Plant Science, Agrifood Research and Technology Centre of Aragon (CITA), Avda. Montañana 930, 50059 Zaragoza, Spainjnavarroro@cita-aragon.es (J.N.-R.); 3 Agrifood Institute of Aragon-IA2 (CITA-University of Zaragoza), 50013 Zaragoza, Spain

**Keywords:** extrusion, total phenols, antioxidant capacity, texture, fatty acid profile

## Abstract

Hemp seeds (HSs) are a rich source of essential fatty acids, proteins, and antioxidant compounds, making them an attractive ingredient for the food industry. This work studies the viability of enriching corn extrudates with hemp seeds, specifically to improve their fatty acid profile and phenolic content, thereby enhancing the nutritional value of the snack. Extrudate formulations with different concentrations of HSs (up to 12.5%) were evaluated, and the physicochemical, textural, and antioxidant properties of the resulting products were analyzed. The results showed that increasing the HS concentration improved the lipid profile of the products, raising the content of unsaturated fatty acids to 75.6% in the snack fortified with 12.5% of HSs and lowering the proportion of saturated fatty acids. This may reduce the risk of cardiovascular diseases compared with corn extrudates. The total phenolic content of the snacks and their antioxidant capacity also increased linearly with the increase of HSs in the formulation. A reduction in specific mechanical energy during extrusion was also observed, attributed to the higher fat content, which facilitates the lubrication of the process. At the physicochemical level, the HS-enriched snacks showed improvements in texture. These snacks were softer, reducing the hardness of the corn snack while maintaining crunchiness. They were even more stable due to a lower water content. The fortification of snacks with hemp seeds provides consumers with a healthier option, while maintaining the appealing crunchy texture and visual appearance regardless of some changes in their color attributes.

## 1. Introduction

Industrial hemp (*Cannabis sativa* L.) contains less than 0.2–0.3% of delta-9-tetrahydrocannabinol (THC) and has been traditionally cultivated to obtain fibers, paper, and grain as a coproduct for animal feed. The development of other natural fibers and synthetic polymers, as well as the abuse of chemotypes with high THC content and narcotic effects, which led to taxation by the administration, finally denigrated this crop in the last century [1,2]. However, many farmers are currently interested in the reintroduction of hemp cultivation in Southern Europe. According to the European Industrial Hemp Association (EIHA), this sector is experiencing an upward trend, and in 2018, the hemp cultivation area in Europe was 50,081 ha [3]. Industrial hemp is a multipurpose crop, with opportunities in several industries from construction to textile to food and beverage applications, whose market share is rising [2,4].

Hemp seed composition makes it an interesting raw material for the food industry due to its nutritional, technological, and organoleptic properties [2,4,5]. It is composed of 23–35% fat [1,6] and 20–25% protein, mainly edestin and albumin, with a desirable content of essential amino acids, comparable to other plant proteins of high nutritional value [5,7,8,9]. Hemp seeds also contain 20–38% carbohydrates, mostly accounting for insoluble fiber [8], bioactive compounds such as terpene hydrocarbons, sterols, polyphenols, tocopherols [10,11], and carotenoids, as well as a low amount of antinutritional constituents (condensed tannins, cyanogenic glycosides, and saponins) [1,4,9]. Besides whole seeds, other products containing hulls or dehulled seeds, seed cake, seed oil, and seed protein isolates can be found in the market [4]. Hemp seed is an emerging ingredient in the food industry, and researchers are still conducting studies to evaluate its techno-functionality to develop new products like bakery [12,13,14], beverages, plant-based meat, and confectionary and snacks [2,4,15,16]. However, most studies consider other raw materials or byproducts derived from hemp seed preprocessing, such as cold pressing, instead of whole seeds [10,17,18,19] and mainly focus on the characterization of the protein fraction and its use as a food ingredient [5,9,14,20], neglecting the lipidic fraction.

On the other hand, Rusu et al. (2021) [12] performed a complete chemical analysis of the seed flour from two hemp varieties and compared them with wheat flour, concluding that the former presented high nutritional quality, especially regarding fatty acid and amino acid composition. Hemp seed flour might be used in many food processes. Therefore, they reinforced a wheat bread with hemp flour, improving its nutritional properties due to a higher content of essential fatty acids, amino acids, minerals, and fiber [13]. However, producers should note that these components also affect the rheological and textural behavior of the final product [14,16]. Thus, traditional products should be fortified with a concentration of hemp seeds that also meet the technological requirements.

Although further research is needed to correlate health benefits with the consumption of hemp-based food, there seems to be an increasing acceptance of edible seeds by consumers, mostly because of the awareness of their health-promoting effects [2,11,21]. Hemp seed composition is genetically and environmentally mediated, but it is characterized by a high polyunsaturated fatty acid (PUFA) content, regardless of variety or crop location, with linoleic (LA, C18:2n-6), α-linolenic acid (ALA, C18:3n-3), and oleic (OA, C18:1n-9) being the major unsaturated fatty acids [1,6,7,12]. These are also the predominant FAs in hemp seed oil and flour [22]. Thus, food products containing hemp seeds may be a valuable source of C18:2n-6 and C18:3n-3, which are classified as essential fatty acids, which may contribute to the proper functioning of human metabolism. As lipid quality indicators, hemp seeds should be considered valuable edible oilseeds because of the proportion of PUFA/SFA and the n-6/n-3 fatty acids (3:1 to 5:1), which help lower the atherogenicity and thrombogenicity indexes [7,22]. Moreover, hemp seed oil and flour have been shown to lower blood cholesterol levels, decrease body weight gain, and reduce epididymal and perirenal adipose tissue, suggesting that both products may be used to prevent obesity [2]. The content of microelements such as K or Mg present in hemp seeds is comparable to that found in flaxseeds, hazelnuts, or walnuts and might exert a cardioprotective effect [12].

Regardless of the susceptibility to oxidation of the fatty acid profile, hemp seed and its oil are considered oxidatively stable due to the presence of tocopherols in the unsaponifiable fraction and phenolic compounds, which contribute to preventing lipid peroxidation [1,11]. Indeed, tocopherols are present in higher amounts in the oil (562.8–971.3 mg/kg) because of their non-polar nature and the extraction process, but several studies report that polyphenols predominate in the seed (770–51,600 mg/kg). These authors also observed a significant correlation between total phenolic content (TPC) and antioxidant activity in whole hemp seed extracts and a weak correlation between tocopherol and antioxidant capacity [1].

The market share of hemp seed and hemp-based food will continue to grow steadily [2,21], and new products might be required. However, Burton et al. (2022) [4] highlighted that there are still some issues that need further attention to build a consistent supply chain and strengthen this market opportunity. Some of them are related to breeding, postharvest handling, and seed processing, particularly regarding the presence of non-allowed constituents in food products. On this subject, it is worth noting that regulation [23] established a maximum permissible THC content of 3 mg/kg in whole hemp seed, milled hemp seed, or in its flour. However, cannabinoids accumulate essentially in the inflorescences and leaves and are absent in roots and seeds; they are not naturally present in the latter because of cross-contamination during harvesting or seed cleaning [1]. There are also dietary exposure assessments stating that hemp-derived foods cause no psychoactivity, allergenicity, or other toxic effects due to their THC content [2]. Moreover, other issues are related to testing applications of hemp seed in traditional food, as well as in emerging market niches [4].

Appetizers are consumed worldwide, and within this category of food products, research in recent years has focused on the nutritional improvement of corn-based extruded snacks by adding novel raw materials from plants or food industry byproducts [24,25]. Extruded corn snacks are trending due to their excellent expansion and texture properties, as well as the capacity of the process to include health benefits [26]. Using extrusion to process bioactive mixtures can achieve not only nutritional improvement but also calorie decrease and inhibition of starch digestion [27].

Considering all this information, hemp seeds might be exploited as an ingredient to obtain potentially functional foods, but more studies must be conducted to reach a balance between technological requirements and quality standards. Previous studies have characterized the proximal composition and focused on the fat profile of different varieties of hemp seed [6] in order to select an appropriate variety to formulate an innovative food product. Thus, considering the high nutritional value of hemp seeds and the need for continuous innovation to obtain easily consumed and ready-to-eat products, the aim of this work is to formulate and elucidate the technological viability of a conventional snack fortified with hemp seeds of the Futura 75 variety and to evaluate the properties and functionality of the final product.

## 2. Results and Discussion

### 2.1. Extrusion Process

The parameters of the extrusion process of corn snacks fortified with P, T_1_, and T_2_ are presented in Table 1. The two temperatures recorded increased significantly (*p* < 0.05) with the incorporation of HSs in the mixtures to be extruded. While the pressure increased with the incorporation of low concentrations of HSs, from 7.5% HS onward, it softened and reached the initial values. Table 1 also includes the specific mechanical energy (SME) mean values. SME is a variable of the extrusion process that simplifies the variables involved in extrusion, according to different authors [28]. SME is defined as the mechanical energy dissipated as heat within the material per unit mass of the material, expressed in J/g [28]. It was calculated according to Logi et al. [29]. The SME values decreased significantly (*p* < 0.05) with the addition of HSs, decreasing the heat dissipated per unit of extrudate produced. Hemp seeds have a higher fat content compared to corn, with 23–30% fat in hemp seeds [6] compared to 3–4% in corn flour [30]. This marked difference in composition reduced SME. Other studies indicate that increasing the fat content in the mixture causes a decrease in SME because the presence of higher lipids causes lubrication of the extruder barrel [31]. In addition, the incorporation of HSs in the mixtures significantly increased (*p* < 0.05) the water loss (W_L_) during the extrusion process, as shown in Table 1. The higher fat content in HS mixtures and the high extrusion temperatures cause a frying effect, which leads to increased water evaporation. The water content of the mixtures before extrusion was around 11%. After extrusion, the control sample (0 HS %) reduced its water content to 9%, while the HS samples contained between 6 and 5% water. This made the HS samples more stable, as they had less water content in their composition.

**Figure 1:**
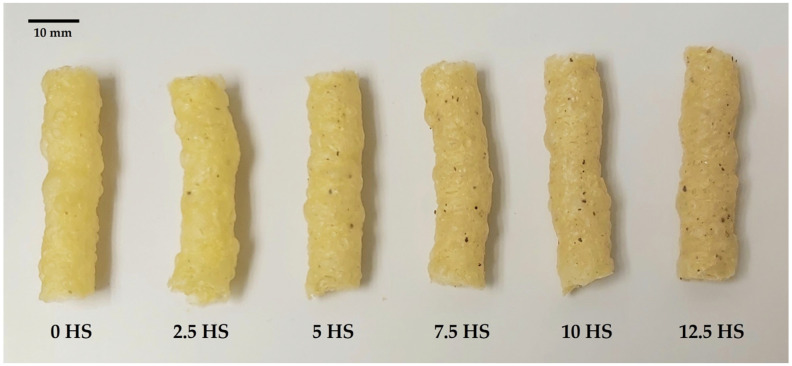
Appearance of the studied extrudates with different concentrations (0, 2.5, 5, 7.5, 10, and 12.5%) of hemp seeds (HSs).

Figure 1 shows the differences between the samples studied. The appearance of the samples is excellent; visually, the color is attractive, and the main difference from the 0 HS sample is the mottling caused by the addition of HSs, which is more pronounced at higher concentrations. There are no major differences in the shape and diameter of the snack.

### 2.2. Total Phenols and Antioxidant Capacity of Extrudates

There are important differences in phenol content among hemp seed varieties, but all of them have a high phenol content, mainly located in the hull [18]. Hemp seeds contain phenolic acids such as vanillic, caffeic, sinapic, ferulic, syringic, and benzoic, and flavonoids such as rutin, naringenin, and quercetin [32]. Table 2 shows the total phenol content (TP) of the extrudates obtained. The incorporation of HSs significantly increased (*p* < 0.05) the TP content of the snacks, and this increase was proportional to the percentage of HSs added, as observed in other hemp-blended products [15,17]. The use of 12.5 HS% approximately doubled the TP content of the 0HSE. In the same way, the antioxidant capacity (AC) of the samples increased significantly (*p* < 0.05) as the percentage of HS increased. In the case of AC, the addition of 12.5 HS% to the mixture almost tripled the value of the 0HSE. Pearson correlations were established among HS %, TP, and AC. The correlation coefficients HS%-TP (0.9713) and HS%-AC (0.9970) were found to be significant (*p* < 0.05) and positive. Besides the HS content in the snacks, extrusion liberates free phenolics from the food matrix, which are bound to other macromolecules, playing a significant role in TP and AC responses [18,19] and probably improving the bioavailability of phenolic compounds. In addition, a significant (*p* < 0.05) Pearson’s coefficient of 0.9641 was obtained for TP-AC. Other studies in different matrices have also observed strong correlations between phenolic compounds and antioxidant capacity [33]. However, hemp seeds also contain tocopherols, sitosterol, and phytol [1,10], which may contribute to the antioxidant activity of the final product.

### 2.3. Fatty Acid Profile of Extrudates

Hemp seed is considered one of the most nutritionally complete food sources due to its high nutritional value [1]. It usually contains 25–35% lipids with a unique and perfectly balanced fatty acid (FA) composition [1,6,7], similar to the results obtained for the Futura 75 variety used in this study, which contains 23 g of fat per 100 g of whole seeds [6]. Table 3 shows the mean values and standard deviation of the lipid content and fatty acids detected in 0HSE and the extrudates enriched with 7.5 and 12.5% HSs. Lipid content increased significantly (*p* < 0.05) with the increase in % HS due to the higher fat content of the hemp seeds compared to corn. The content of each fatty acid studied in the extrudates increased with the addition of HSs, except for C14:1n-5, which decreased, and C12:0, C14:0, and C21:0, which remained unchanged in 0HSE. The rest of the fatty acids, especially the unsaturated fatty acids, increased significantly (*p* < 0.05) with the addition of HSs compared to 0HSE. Table 3 shows that the major FA in 0HSE is palmitic acid (C16:0), saturated, in comparison with the HS-enriched snacks, whose major FA is C18:2n-6, polyunsaturated. In addition, 7.5HSE and 12.5HSE had almost four and seven times more C18:2n-6 than 0HSE, respectively. C18:2n-6 is the major fatty acid in hemp seeds [6]; this fatty acid is approximately 55% of the total fatty acids in hemp seeds [1,4,7,34,35]. The next major fatty acid in hemp seed is C18:3n-3 [6]. This is reflected in the significant (*p* < 0.05) increase of this FA in the HS snacks. Since the concentration of this FA in 0HSE is low, 7.5HSE and 12.5HSE have 14 and 30 times more C18:3n-3 than 0HSE, respectively. C18:3n-3 is a precursor to other important omega-3 fatty acids, such as eicosapentaenoic acid (EPA, C20:5n-3) and docosahexaenoic acid (DHA, C22:6n-3). Studies have shown that C20:5n-3 and C22:6n-3 are important for proper fetal development, including neuronal, retinal, and immune functions. EPA and DHA may affect many aspects of cardiovascular function, including inflammation, peripheral artery disease, major coronary events, and anticoagulation [36]. The addition of HSs in the mixtures to be extruded generates snacks with unsaturated FAs that are not detected in corn-only extrudates, such as γ-linolenic acid (C18:3n-6), stearidonic acid (SDA, C18:4n-3), and eicosadienoic acid (EDA, C20:2n-6). SDA was undoubtedly provided by the fortification with hemp seed because it is a common PUFA in other plant species and has been previously reported in different hemp varieties, being a representative compound of the n-3 lipid fraction [7,22]. EDA is a rare, naturally occurring n-6 polyunsaturated fatty acid. It is elongated from C18:2n-6 [37] and was associated with lower homeostasis model assessment-estimated insulin resistance and incident type 2 diabetes in participants from the Multi-Ethnic Study of Atherosclerosis cohort [38]. The incorporation of HSs also markedly, linearly, and significantly increased (*p* < 0.05) the content of unsaturated fatty acids such as palmitoleic acid (C16:1n-6), oleic acid (C18:1n-9), cis-vaccenic acid (C18:1n-7), and eicosenoic acid (C20:1). For all these reasons, HS-enriched snacks represent a considerable improvement in the fat profile of the corn extrudates currently offered by the industry and can be considered healthy snacks. The intake of 100 g of the extrudate with 12.5% HSs would meet the RDAs for linoleic acid and α-linolenic acid recommended by AESAN and EFSA [39].

Table S1 summarizes the relative FA content as a percentage of the total FAs in the extrudates studied. The results indicate significant (*p* < 0.05) changes in the FA composition with increasing levels of hemp seed enrichment. A consistent decrease in the levels of saturated fatty acids, such as C12:0, C14:0, C15:0, C16:0, and C18:0, is observed with increasing hemp seed percentage in the extrudates. For instance, C16:0 dropped from 33.3% in 0HSE to 14.9% in 12.5HSE. This indicates that hemp seed enrichment effectively reduces the relative abundance of SFAs, contributing to a potentially healthier lipid profile and reducing the risk of various diseases [11]. A significant (*p* < 0.05) increase in key MUFAs, such as C18:1n-9 and C18:1n-7, is evident. C18:1n-9, a beneficial fatty acid associated with improved cardiovascular health, increased from 10.3% in 0HSE to 14.6% in 12.5HSE. Similarly, C20:1n-9 also showed a notable increase, although at lower levels. The most pronounced changes were observed in C18:2n-6, which increased significantly (*p* < 0.05) from 26.5% in 0HSE to 48.6% in 12.5HSE. Additionally, C18:3n-6 and C20:2n-6 emerged in the enriched samples, further increasing the n-6 PUFA content. A marked increase in C18:3n-3, a critical omega-3 fatty acid, was noted—from 1.17% in 0HSE to 9.32% in 12.5HSE. This reflects the high n-3 PUFA content of hemp seeds. Additionally, the appearance of C18:4n-3 in the enriched samples highlights the contribution of hemp seeds to diversifying the n-3 fatty acid profile. The increased levels of n-3 fatty acids, particularly C18:3n-3, suggest that hemp seed enrichment may help achieve a more favorable n-6/n-3 ratio, which is an important factor for anti-inflammatory and health-promoting effects [40]. Minor fatty acids, such as C14:1n-5 and C22:1n-9, and very long-chain SFAs (e.g., C23:0 and C24:0) showed slight variations, but their contributions to the overall lipid profile remained minimal.

**Figure 2:**
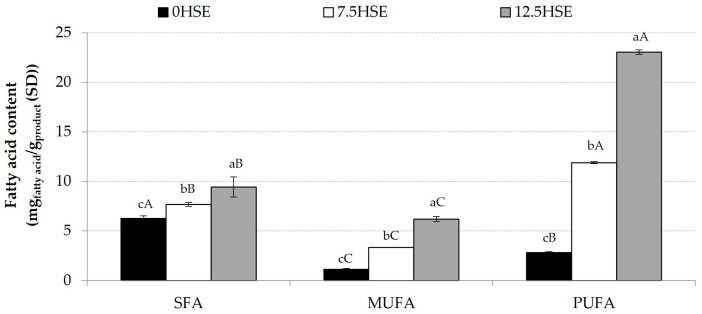
Fatty acids (FAs) per group. SFA: saturated fatty acid, MUFA: monounsaturated fatty acid, and PUFA: polyunsaturated fatty acid. The same small letter in superscript indicates homogeneous groups established using ANOVA (*p* < 0.05) among samples. The same capital letter in superscript indicates homogeneous groups established using ANOVA (*p* < 0.05) among FA groups.

After evaluating the fat profile of the samples studied, the global results by FA group are presented in Figure 2: saturated fatty acids (SFAs), monounsaturated fatty acids (MUFAs), and polyunsaturated fatty acids (PUFAs). The last two groups are united into unsaturated fatty acids (UFAs). The use of HSs in the formulation of snacks increased the total FA content. However, it is clearly observed that SFAs increased far less than MUFAs and, in turn, far less than PUFAs. The 12.5HSE sample had the highest PUFA and MUFA content. The percentage of SFAs and UFAs compared to total FA varied markedly and significantly (*p* < 0.05) with the addition of HSs. While in 0HSE, SFA content is 61.3%, in 12.5HSE, UFA content is 75.61%. Furthermore, 59.6% of the total FA is PUFA in this snack. These results reaffirm the improvement in the nutritional and functional value of HS-enriched extrudates, making them healthier snacks.

There are indices widely used to assess the potential health effects of dietary fatty acids, particularly in relation to cardiovascular risk factors and disease development. Evaluating the atherogenicity (AI) and thrombogenicity (TI) indices offers valuable insights into the potential impacts of individual fatty acids on human health, particularly concerning the risk of developing atherosclerosis, blood clots, atheromas, and thrombi formation [41,42]. AI serves as an early indicator of accelerated atherosclerosis and aids in understanding the various inflammatory pathways involved, while TI reflects the propensity for blood clot formation and the risk of cardiovascular diseases [41,43]. AI and TI were calculated according to Ulbricht and Southgate (1991) [41] using the following equations:

$$AI=\frac{C12:0+4\times C14:0+C16:0}{\sum MUFA+\sum PUFAn-6+\sum PUFAn-3}$$

$$TI=\frac{C14:0+C16:0+C18:0}{0.5\sum MUFA+0.5\sum PUFAn-6+3\sum PUFAn-3+\frac{\sum PUFAn-3}{\sum PUFAn-6}}$$

Table 4 shows the AI and TI of extrudates without hemp seeds (0HSE) and with hemp seeds (7.5HSE and 12.5HSE). The inclusion of 7.5% hemp seeds (7.5HSE) resulted in a significant (*p* < 0.05) reduction in both AI and TI compared to the control, indicating the beneficial modulation of the lipid profile. Further enrichment to 12.5% (12.5HSE) yielded even lower indices, suggesting the dose-dependent effect of hemp seed incorporation on improving the nutritional quality of the extrudates. The observed trends underscore the potential of hemp seed-enriched extrudates to serve as functional foods aimed at mitigating cardiovascular risk factors.

### 2.4. Physical, Textural, and Optical Properties of Corn Extrudates

Although nutritional and functional improvement has been fully proven with the results presented for FA, TP, and AC, it is necessary to study the effect of HS addition on the physical, textural, and optical properties of the extrudates, as these are key properties for product acceptance. Table 5 shows the values of water content (x_w_), water absorption index (WAI), and water solubility index (WSI) of the extrudates. The incorporation of HSs in the mixtures made it possible to obtain extrudates with lower x_w_, and therefore safer, because the lower the water content in food, the lower the possibility of microorganism development. This trend of greater stability is also reflected in the WSI, which is lower in the samples with HSs, agreeing with the results obtained by Norajit et al. (2011) [15]. WSI indicates the amount of small molecules solubilized in water, causing molecular damage in the process [44]. Therefore, HSs in samples reduces the risk of possible molecular damage caused by water-solubilized molecules. HS reduces the solubilization of matrix components during extrusion, mainly by showing less corn starch degradation and the formation of soluble fragments. WAI indicates the amount of water immobilized by the extrudate [45]. Samples containing HSs showed an oscillatory behavior in WAI values with increasing % HS, simulating a sinusoidal function. The sample with 7.5% HSs showed significantly similar values to 0HSE than the rest.

Table 5 also contains the values of expansion index (SEI), porosity (ε), and bulk density (ρ_b_). Expansion occurs due to the pressure drop caused by the passage of the melt from elevated pressure to atmospheric pressure as it leaves the sealed matrix [46]. The density of extrudates is a general property that indicates overall expansion and changes in material parameters and cell structure. The bulk density of extrudates describes the degree of swelling the material undergoes as it leaves the extruder and, therefore, considers expansion in all directions. However, the SEI only considers expansion in the direction perpendicular to the extrusion flow [47]. The SEI values decreased linearly and significantly (*p* < 0.05) after the addition of 5% HSs. However, the decrease is not very marked compared to other corn snacks enriched with alfalfa [25] or nettle [48]. Extruded rice-hemp products also expanded less with increasing hemp seed powder, which may be related to fiber content and the rupture of cell walls by these macromolecules [15]. ρ_b_ showed oscillatory behavior with increasing HS content in the snacks. The same trend was observed for WAI, with the sample with the 7.5% HS sample showing no significant (*p* > 0.05) differences with 0HSE. During extrusion processing, pores and voids develop, and they are responsible for the characteristic extrudate structure. Highly expanded extruded materials present a porous structure [46]. To evaluate the volume of air occluded in the extrudates, ε values are studied. The ε of the HS-enriched samples showed oscillatory behavior with increasing HS concentration, in contrast to ρ_b_. The 7.5HSE sample had the most similarities with respect to 0HSE, especially in ε, ρ_b_, and WSI, as they did not show significant differences. Concentrations higher than 7.5% HS did not show marked differences from 0HSE and reduced the x_w_ and WSI to a greater degree, providing stability to the sample.

Texture is one of the most important characteristics of extruded snacks that help to improve the quality of food products [49]. The texture of the extruded product is highly dependent on the composition of the raw material used for extrusion [46]. The different texture parameters studied (W_c_, F_p_, F_s_, N_sr_, and N_0_) are shown in Table 6. W_c_ describes the work required to fracture one pore or a group of pores and represents the sensory parameter of fracturability. The F_p_ and F_s_ of extruded products are associated with the sensory perception of hardness during chewing. N_sr_ describes the number of fracture events during puncture, and N_0_ is the number of fractures along the puncture assay; both are related to the crunchiness of the snack [50]. Looking at these parameters (N_sr_ and N_0_), the addition of HSs in increasing concentrations did not affect the crispiness of the snack. The values of N_sr_ and N_0_ showed no significant (*p* > 0.05) differences among the 0HSE and HS-enriched samples. But when 7.5% HS or higher concentrations were added, W_c_ was reduced and less energy was expended to achieve crispness in the mouth. HS snacks are softer, reducing the hardness of the corn snack (F_p_ and F_s_) while maintaining crunchiness. A similar behavior was described for corn snacks enriched with carob beans [51].

Table 7 shows the relationship between the FA content (total and by groups) and W_L_, SME, SEI, ε, and W_c_. There are high and significant (*p* < 0.05) correlations between FA and the studied variables. An increase in the ToFA of the snacks leads to an increase in W_L_ and a decrease in SME, SEI, ε, and W_c_. The highest Pearson coefficients are those relating the studied variables to the PUFA group.

Because color is a relevant property in consumer acceptance, one of the main shortcomings of adding novel plant ingredients to food formulations lies in the potential unpleasant organoleptic properties they may impart to the original product. However, several studies report an improvement in this attribute after incorporating hemp-based ingredients into different food products, such as meat balls or pasta [2]. Table 8 shows the values of the color coordinates (L*, a*, b*, C*, and h*) of the extrudates with the % HS studied (0–12.5). The total color differences (ΔE) are also included. The increase in HS concentration in the snacks results in a significantly (*p* < 0.05) greater ΔE, mainly due to the variation in L*, which follows the same decreasing trend as in other hemp-based products [14,17], probably attributed to chemical changes in polyphenols and chlorophyl during processing [2]. Considering the visual appearance of the samples, the authors suggest that the changes in snack lightness are caused by the mottling produced by HS addition, as indicated above (Section 2.1). Despite the decrease in L*, the snacks fortified with HSs have an attractive appearance (Figure 2), similar to breads containing hemp-based ingredients regardless of the visual differences with control bread [14,52]. The oscillatory behavior in redness (a*) with increasing HS concentration agrees with the results observed by Feng et al. (2022) [17] when incorporating hemp seed cake into potato chips. It is suggested that, to some extent, the greater protein content of products fortified with HSs may lead to non-enzymatic reactions with sugars, producing brown pigments [17]. Hemp seed cake also increases b* [17], but this upward trend did not occur in snacks enriched with different proportions of HSs. On the contrary, a higher b* value was obtained in control corn snacks, which steadily decreased with the addition of HSs. This was probably produced by the substitution of the yellowish color given by corn with the characteristic brownish color of hemp seeds.

## 3. Materials and Methods

### 3.1. Raw Materials, Formulations, and Extrusion Process

Maicerías Españolas S.L. (Valencia, Spain) supplied corn grits. Hemp seeds (HSs) of Futura 75 variety were supplied by Trichome Pharma (Madrid, Spain) for research purposes only. The HSs were ground (Minimoka, Taurus, Lleida, Spain) to obtain powder.

The corn grits were mixed manually using a whisk, with increasing HS powder percentages of 2.5, 5, 7.5, 10, and 12.5%, to produce the extrusion mixtures.

**Figure 3:**
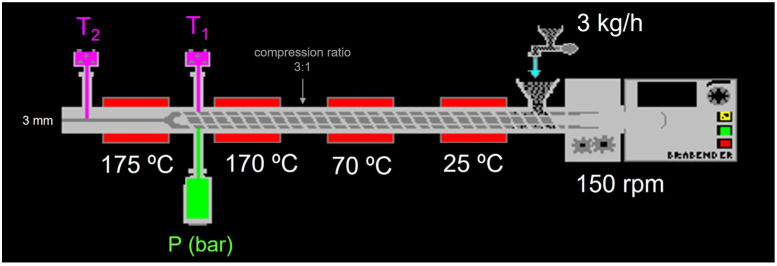
Scheme of conditions used in the extruder.

Extrusion was performed using a single-screw laboratory extruder with a barrel diameter of 19 mm and a length–diameter ratio of 25:1 (Kompaktextruder KE 19/25; Brabender, Duisburg, Germany). Figure 3 shows the conditions during extrusion. Screw speed, motor torque, melt pressure (P), and barrel temperatures (T_1_ and T_2_) were monitored using Extruder Winext software (version 4.4.3) (Brabender). The extrudates were cooled at 23 °C and sealed in plastic bags for further analysis.

### 3.2. Total Phenol (TP) and Antioxidant Capacity (AC) Determination

TP was determined based on the Folin–Ciocalteu method. Phenolic compound extraction consisted of mixing 0.5 g of the sample and 4 mL methanol for 1 min; the resulting mixture was transferred to an ultrasonic bath (Ultrasons H-D, J.P. Selecta, Barcelona, Spain) for 5 min. Then, the samples were centrifuged (10,000 rpm, 10 min, 4 °C) to obtain the supernatant using Eppendorf Centrifuge 5804 R (Hamburg, Germany). For determination, 15 mL of distilled water and 1.25 mL of Folin–Ciocalteu reagent (Sigma-Aldrich, Steinheim, Germany) were added to 250 μL of the supernatant. The samples were mixed and allowed to stand for 8 min in darkness before adding 3.75 mL of 7.5% sodium carbonate aqueous solution. Water was added to adjust the final volume to 25 mL. The samples were allowed to stand for 2 h at room temperature before measurement. Absorbance was measured at 765 nm using a UV-3100PC spectrophotometer (VWR, Leuven, Belgium). The total phenolic content was expressed as mg of gallic acid equivalents (GAE) (Sigma-Aldrich, Steinheim, Germany) per 100 g. The analysis was carried out in triplicate.

AC was assessed using the free radical scavenging activity of the samples evaluated with the stable radical 2,2-diphenyl-1-picryl-hydrazyl-hydrate (DPPH) following Igual et al.’s [33] methodology in triplicate. The samples were mixed with methanol. The homogenate was centrifuged (10,000 rpm, 10 min, 4 °C) to obtain the supernatant. Then, 0.1 mL of supernatant was added to 3.9 mL of DPPH• (0.030 g/L, Sigma-Aldrich, Steinheim, Germany) in methanol. A UV-3100PC spectrophotometer (VWR, Leuven, Belgium) was used at an absorbance of 515 nm. The results were expressed as milligrams of Trolox equivalents (TE) per 100 g.

### 3.3. Lipid Content and Fatty Acid Profile Determination

The total fat content was determined gravimetrically using acid hydrolysis and Soxhlet extraction following a procedure similar to AOAC 922.06 [53]. The fatty acid (FA) composition was determined using the gas chromatography technique (Bruker Scion 436, Bruker, Billerica, MA, USA) after lipid extraction, and FA methyl ester synthesis performed according to Rufino-Moya et al. (2022) [54]. FA composition was measured in the control extrudates without hemp seed (0HSE), extrudates with 7.5% hemp seed enrichment (7.5HSE), and extrudates with 12.5% hemp seed enrichment (12.5HSE). Separation was performed in an SP-2560 capillary column (100 m × 0.25 mm × 0.2 μm) (Supelco, Saint Louis, MO, USA) after 1 μL injection with a split ratio of 1:125 using helium as carrier gas at a flow of 1.5 mL/min. The injector was set at 280 °C and the oven temperature was set at 125 °C for 15 min and increased at a rate of 5 °C/min to 190 °C. The operating conditions of the flame ionization detector were 280 °C and 30 and 300 mL/min of hydrogen and combustion gas, respectively. C19:0 was used as an internal standard in the extraction of FA methyl esters (FAMEs), and identifications were based on FAME retention times, which were compared with those of the standard mixtures GLC-(401, 463, 532, 538, 642, 643), C18:1n-7, and tC18:1n-7 (Nu-Chek Prep, Elysian, MN, USA) or with relative retention times found in the literature [55,56] in the case of C18:4n3. Quantification was performed as described in ISO 12966-4:2015 [57].

### 3.4. Determination of Physicochemical Properties

Water content (x_w_) (g/100 g) was determined using vacuum oven drying at 105 °C until a constant weight was achieved [58] for the mixtures and extruded samples. The samples were analyzed in triplicate.

One of the most interesting properties of extruded products is expansion. This can be measured by different parameters, among which the surface expansion index (SEI) of the die and bulk density stand out (ρ_b_). While ρ_b_ considers expansion in all directions, SEI considers expansion only in the direction perpendicular to the extrudate flow [47]. SEI was calculated as the quotient between the square of the measured extrudate diameters and the square of the die diameter [49]. In total, 20 diameters of extruded pieces were measured for each sample with an electronic Vernier caliper (Comecta S.A., Barcelona, Spain). For ρ_b_ determination, measurements were taken 10 times, where the diameter and height of cylinders were measured with an electronic Vernier caliper (Comecta S.A., Barcelona, Spain), and the samples were weighed with a precision scale (±0.001 g) (Mettler Toledo, Greifensee, Switzerland).

The porosity (ε), percentage of air volume related to the total volume, was calculated from the true (ρ) and bulk (ρ_b_) densities according to Igual et al. [24]. The ρ of the extruded products was determined in triplicate using a helium pycnometer (AccPyc 1330, Micromeritics, Norcross, GA, USA).

To evaluate the hydration properties, the water absorption index (WAI) and water solubility index (WSI) were used. The WSI and WAI were determined using the method of Singh et al. [59] and calculated according to Uribe-Wandurraga et al. [21]. The swelling index (SWE) also was measured using the bed volume technique. The bed volume was recorded and expressed as mm of swollen sample per g of dry initial sample [24].

Texture properties were measured using puncture tests with a TA-XT2 Texture Analyzer (Stable Micro Systems Ltd., Godalming, UK) and Texture Exponent software (version 6.1.12.0). A 2 mm diameter cylinder was used, and the crosshead speed was kept at 0.6 mm/s [25]. From the force–time curve, the area under the curve plot, which represented work done for a time of displacement of the puncturing device, was obtained from the extrudates (8 times). The force drop of each peak was also obtained, and it represented the local resistance of cell walls. The number of peaks (No) were also recorded [60]. These parameters were used to calculate the average puncturing force (Fp), average specific force of structural ruptures (Fs), spatial frequency of structural ruptures (Nsr), and crispness work (Wc) according to Igual et al. [30].

Color was determined in the powdered samples. Flours and extrudates were ground and placed in a cup specially designed for this purpose. Color was measured using a Minolta spectrophotometer CM-3600d (Tokyo, Japan). CIE*L*a*b* color coordinates were determined considering standard light source D65 and a standard observer at 10° for the mixtures and extrudates (8 times). Hue (h*) and chroma (C*) color attributes were calculated from the CIE*L*a*b* color coordinates. The total color differences of the extrudates with HSs (ΔE) were calculated relative to the 0HSE sample [25].

### 3.5. Statistical Analysis

Analysis of variance (ANOVA), with a confidence level of 95% (*p* < 0.05), was applied using Statgraphics Centurion XVII, version 17.2.04, to evaluate the differences among the samples. Fisher’s least significant difference procedure was used to discriminate between means. Correlation analyses were performed with a significance level of 95% (Statgraphics Centurion XVII, Statgraphics Technologies, Inc., The Plains, VA, USA).

## 4. Conclusions

The incorporation of hemp seeds into corn extrudates offers a promising strategy to improve the nutritional value of conventional snacks by increasing polyphenols, antioxidant capacity, and unsaturated fatty acids, especially those that are essential, such as linoleic (C18:2n-6) and linolenic (C18:3n-3) acids. There is no need to incorporate individual ingredients from separate seed macronutrients, and it is feasible to manufacture snacks from whole hemp seeds, providing consumers with a healthier option due to the lower proportion of saturated fatty acids, which may reduce the risk of cardiovascular disorders compared with a traditional corn-based snack. This enrichment not only optimizes the nutritional value of the product but also improves its physicochemical characteristics, such as texture and stability, while maintaining the appealing crunchiness and attractive visual appearance despite some changes in color attributes. In a market increasingly focused on functional food and health benefits, these hemp-fortified products can stand out for their nutritional–functional potential and excellent sensory characteristics.

## Figures and Tables

**Table 1:** Mean values (and standard deviations) of parameters of the extrusion process of fortified corn snacks.

HS %	P (Pa)	T_1_ (°C)	T_2_ (°C)	SME (J/g)	W_L_ (g_w_/g_db_)
0	131 (16) ^cd^	97.7 (0.5) ^e^	173.6 (1.7) ^e^	640.7 (0.6) ^a^	0.025 (0.006) ^c^
2.5	191 (7) ^a^	173.4 (0.5) ^d^	179.4 (0.5) ^c^	507.4 (1.2) ^e^	0.068 (0.003) ^b^
5	167 (11) ^b^	174.7 (1.2) ^c^	179.7 (0.5) ^bc^	580.9 (1.3) ^c^	0.067 (0.002) ^b^
7.5	139 (6) ^c^	179.1 (1.2) ^b^	180.0 (0.2) ^bc^	578 (3) ^c^	0.068 (0.004) ^b^
10	130 (2) ^d^	181.4 (0.5) ^a^	180.3 (0.4) ^ab^	612 (2) ^b^	0.073 (0.002) ^ab^
12.5	132 (2) ^cd^	182.0 (0.2) ^a^	181.0 (0.2) ^a^	568.4 (0.9) ^d^	0.075 (0.003) ^a^

The same letter in superscript within a column indicates homogeneous groups established using ANOVA (*p* < 0.05). P: melt pressure; T_1_, T_2_: barrel temperatures; SME: specific mechanical energy; W_L_: water loss.

**Table 2:** Mean (SD) values of total phenols (TP [mgGAE/100g_extrudate_]) and antioxidant capacity (AC [mgTE/100g_extrudate_]) of the studied extrudates enriched with different concentrations (0, 2.5, 5, 7.5, 10, and 12.5%) of hemp seeds (HSE).

Sample	TP	AC
0HSE	79.2 (1.2) ^e^	30.58 (0.04) ^f^
2.5HSE	93 (2) ^d^	41.9 (1.2) ^e^
5HSE	97.2 (0.9) ^d^	54.0 (0.5) ^d^
7.5HSE	112 (7) ^c^	60.3 (1.5) ^c^
10HSE	126.9 (1.2) ^b^	73.8 (0.7) ^b^
12.5HSE	147 (3) ^a^	83.4 (0.6) ^a^

The same letter in superscript within a column indicates homogeneous groups established using ANOVA (*p* < 0.05).

**Table 3:** Mean (SD) values of lipid (g/100g_extrudate_) and fatty acid (µg/g_extrudate_) content of control extrudates without hemp seed (0HSE), extrudates with 7.5% hemp seed enrichment (7.5HSE), and extrudates with 12.5% hemp seed enrichment (12.5HSE).

Sample	0HSE	7.5HSE	12.5HSE
Lipid	3.86 (0.08) ^c^	5.42 (0.05) ^b^	6.38 (0.12) ^a^
C12:0	28.7 (1.2) ^a^	28.2 (1.8) ^a^	28 (4) ^a^
C14:0	51.3 (1.8) ^b^	54.2 (1.2) ^ab^	60 (2) ^a^
C14:1n-5	2.9 (0.2) ^a^	1.3 (0.2) ^b^	1.2 (0.4) ^b^
C15:0	6.2 (0.2) ^b^	11.3 (0.2) ^a^	15 (2) ^a^
C16:0	3414 (120) ^c^	4442 (42) ^b^	5777 (214) ^a^
C16:1n-7	7.1 (1.2) ^c^	26 (2) ^b^	49 (4) ^a^
C17:0	12.1 (0.2) ^c^	15.7 (0.2) ^b^	22.2 (1.4) ^a^
C18:0	2719 (14) ^c^	2978 (48) ^b^	3254 (53) ^a^
C18:1n-9	1057 (59) ^c^	3068 (120) ^b^	5665 (215) ^a^
C18:1n-7	49 (3) ^ab^	183 (5) ^b^	365 (14) ^a^
C18:2n-6	2716 (120) ^c^	9927 (143) ^b^	18,823 (555) ^a^
C18:3n-6	- ^c^	229.3 (1.2) ^b^	513 (5) ^a^
C20:0	35 (5) ^c^	113.09 (0.04) ^b^	208 (3) ^a^
C18:3n-3	120 (6) ^c^	169 (41) ^b^	361 (26) ^a^
C20:1n-9	7.9 (1.3) ^c^	50 (3) ^b^	106 (2) ^a^
C18:4n-3	- ^c^	46 (4) ^b^	101 (5) ^a^
C21:0	4.3 (8) ^a^	4.0 (0.3) ^a^	5.7 (0.4) ^a^
C20:2n-6	- ^c^	8.8 (0.3) ^b^	17 (2) ^a^
C22:0	3.5 (1.2) ^c^	24.1 (0.2) ^b^	52 (5) ^a^
C22:1n-9	2.9 (0.3) ^b^	5.8 (0.6) ^a^	6.3 (0.6) ^a^
C23:0	0.6 (0.3) ^b^	3.2 (0.8) ^a^	3.2 (0.8) ^a^
C24:0	11.3 (0.6) ^b^	6 (2) ^b^	14 (2) ^a^

The same letter in superscript within a row indicates homogeneous groups established using ANOVA (*p* < 0.05). Nomenclature of FAs: Number after ‘C’ indicates the number of carbons, number after ‘:’ indicates the number of double bonds, and number after ‘n’ indicates the position of the first double bond from the methyl end of the FA.

**Table 4:** Atherogenicity (AI) and thrombogenicity (TI) indices of control extrudates without hemp seed (0HSE), extrudates with 7.5% hemp seed enrichment (7.5HSE), and extrudates with 12.5% hemp seed enrichment (12.5HSE).

Sample	0HSE	7.5HSE	12.5HSE
AI	0.921 (0.008) ^a^	0.308 (0.002) ^b^	0.207 (0.002) ^c^
TI	2.66 (0.03) ^a^	0.6162 (0.004) ^b^	0.377 (0.007) ^c^

The same letter in superscript within a row indicates homogeneous groups established using ANOVA (*p* < 0.05).

**Table 5:** Mean (SD) values of water content (x_w_), expansion index (SEI), porosity (ε), bulk density (ρ_b_), water absorption index (WAI), and water solubility index (WSI) of extrudates enriched with different concentrations (0, 2.5, 5, 7.5, 10, and 12.5%) of hemp seeds (HSE).

Sample	x_w_ (g_w_/100g)	SEI	ε	ρ_b_ (g/cm^3^)	WAI	WSI
0HSE	8.7 (0.3) ^a^	14.1 (0.4) ^a^	93.7 (0.2) ^a^	0.085 (0.007) ^c^	5.562 (0.003) ^c^	10.72 (0.07) ^a^
2.5HSE	6.0 (0.2) ^b^	14.0 (0.4) ^a^	89.6 (0.5) ^c^	0.114 (0.005) ^a^	6.34 (0.15) ^a^	4.6 (0.4) ^e^
5HSE	5.33 (0.12) ^c^	13.5 (0.3) ^b^	89.83 (0.08) ^c^	0.1058 (0.0014) ^ab^	5.90 (0.03) ^b^	5.80 (0.08) ^d^
7.5HSE	5.6 (0.3) ^c^	12.5 (0.5) ^c^	93.13 (0.12) ^a^	0.084 (0.002) ^c^	5.59 (0.07) ^c^	8.969 (0.014) ^b^
10HSE	4.84 (0.04) ^d^	11.4 (0.2) ^d^	92.09 (0.12) ^b^	0.0998 (0.0012) ^b^	5.90 (0.12) ^b^	7.185 (0.013) ^c^
12.5HSE	4.62 (0.09) ^d^	10.8 (0.3) ^e^	91.60 (0.04) ^b^	0.1043 (0.0006) ^b^	5.94 (0.04) ^b^	7.3 (0.2) ^c^

The same letter in superscript within a column indicates homogeneous groups established using ANOVA (*p* < 0.05).

**Table 6:** Mean (SD) values of crispness work (W_c_), spatial frequency of structural ruptures (N_sr_), average specific force of structural ruptures (F_s_), average puncturing force (F_p_), and number of peaks (N_0_) of extrudates enriched with different concentrations (0, 2.5, 5, 7.5, 10, and 12.5%) of hemp seeds (HSE).

Sample	W_c_ (N*mm)	N_sr_ (mm^−1^)	F_s_ (N)	F_p_ (N)	N_0_
0HSE	0.55 (0.08) ^b^	5.7 (0.6) ^ab^	3.2 (0.5) ^ab^	2.6 (0.3) ^a^	62 (3) ^ab^
2.5HSE	0.76 (0.12) ^a^	4.8 (0.6) ^b^	3.6 (0.5) ^a^	2.4 (0.2) ^a^	57 (2) ^c^
5HSE	0.49 (0.06) ^b^	5.3 (0.6) ^b^	2.6 (0.6) ^b^	1.9 (0.4) ^b^	66 (3) ^a^
7.5HSE	0.30 (0.02) ^c^	5.5 (0.6) ^b^	1.7 (0.3) ^c^	1.4 (0.3) ^c^	66 (3) ^a^
10HSE	0.32 (0.06) ^c^	4.8 (0.6) ^b^	1.5 (0.2) ^c^	1.06 (0.16) ^c^	60 (3) ^bc^
12.5HSE	0.29 (0.04) ^c^	6.5 (0.6) ^a^	1.9 (0.2) ^c^	1.5 (0.2) ^c^	62.3 (0.5) ^ab^

The same letter in superscript within a column indicates homogeneous groups established using ANOVA (*p* < 0.05).

**Table 7:** Pearson correlation coefficients between some physicochemical parameters and fatty acid content of extrudates. Water loss (W_L_), specific mechanical energy (SME), expansion index (SEI), porosity (ε), crispness work (W_c_), total fatty acid (ToFA), saturated fatty acid (SFA), monounsaturated fatty acid (MUFA), and polyunsaturated fatty acid (PUFA).

	W_L_	SME	SEI	ε	W_c_
ToFA	0.8741 *	−0.8908 *	−0.9934 *	−0.9761 *	−0.8334 *
SFA	0.8635 *	−0.8869 *	−0.9878 *	−0.9705 *	−0.8264 *
MUFA	0.8686 *	−0.8846 *	−0.9919 *	−0.9776 *	−0.8263 *
PUFA	0.8768 *	−0.8927 *	−0.9943 *	−0.9762 *	−0.8360 *

* Correlation is significant at 0.05. All data represent the mean of three determinations.

**Table 8:** Mean (SD) values of color coordinates (L*, a*, b*, C*, and h*) and total color differences (ΔE) of extrudates enriched with different concentrations (0, 2.5, 5, 7.5, 10, and 12.5%) of hemp seeds (HSE).

Sample	L*	a*	b*	C*	h*	ΔE
0HSE	83.175 (0.013) ^a^	2.98 (0.02) ^e^	33.488 (0.012) ^b^	33.620 (0.012) ^b^	84.92 (0.04) ^a^	-
2.5HSE	81.325 (0.006) ^b^	3.102 (0.012) ^d^	34.690 (0.008) ^a^	34.830 (0.008) ^a^	84.86 (0.02) ^a^	2.211 (0.007) ^d^
5HSE	78.943 (0.013) ^c^	3.24 (0.02) ^c^	31.873 (0.012) ^c^	32.037 (0.008) ^c^	84.20 (0.04) ^b^	4.54 (0.02) ^c^
7.5HSE	76.455 (0.006) ^d^	3.87 (0.02) ^a^	30.093 (0.012) ^d^	30.340 (0.008) ^d^	82.68 (0.03) ^e^	7.581 (0.012) ^b^
10HSE	74.2 (0.2) ^f^	3.52 (0.03) ^b^	28.3 (0.2) ^e^	28.5 (0.2) ^e^	82.90 (0.11) ^d^	10.4 (0.3) ^a^
12.5HSE	74.680 (0.008) ^e^	2.865 (0.013) ^f^	27.710 (0.018) ^f^	27.858 (0.017) ^f^	84.10 (0.03) ^c^	10.27 (0.02) ^a^

The same letter in superscript within a column indicates homogeneous groups established using ANOVA (*p* < 0.05).

## Data Availability

The raw data supporting the conclusions of this article will be made available by the authors on request.

## Supplementary Materials

The following supporting information can be downloaded at: https://www.mdpi.com/article/10.3390/molecules30061390/s1, Table S1: Mean (SD) values of relative fatty acids content to total FA (%) of control extrudates without hemp seed (0HSE), extrudates with 7.5 % of hemp seeds enrichment (7.5HSE), and extrudates with 12.5 % of hemp seeds enrichment (12.5HSE).
