# Supplementary material for: Fatty Acid Enrichment of Corn Extrudates with Hemp Seeds
Source: Molecules. 2025 Mar 20;30(6):1390. doi: 10.3390/molecules30061390 (PMC11945906; doi:10.3390/molecules30061390)
Supplement: Supplementary file 1 [file molecules-30-01390-s001.zip › molecules-3474806-supplementary.pdf]

Table S1. Mean (SD) values of relative fatty acids content to total FA (%) of control extrudates without hemp seed (0HSE), extrudates with 7.5 % of hemp seeds enrichment (7.5HSE), and extrudates with 12.5 % of hemp seeds enrichment (12.5HSE)

Sample	0HSE	7.5HSE	12.5HSE
C12:0	0.280 (0.09)^{a}	0.123 (0.008)^{b}	0.073 (0.009)^{c}
C14:0	0.501 (0.17)^{a}	0.237 (0.005)^{b}	0.154 (0.006)^{c}
C14:1n-5	0.028 (0.008)^{a}	0.0055 (0.0009)^{b}	0.0031 (0.0012)^{b}
C15:0	0.061 (0.002)^{a}	0.049 (0.003)^{b}	0.038 (0.006)^{b}
C16:0	33.3 (1.2)^{a}	19.4 (0.2)^{b}	14.9 (0.6)^{c}
C16:1n-7	0.070 (0.012)^{c}	0.113 (0.008)^{b}	0.128 (0.012)^{a}
C17:0	0.118 (0.002)^{a}	0.068 (0.002)^{b}	0.057 (0.004)^{b}
C18:0	26.56 (0.14)^{a}	13.0 (0.2)^{b}	8.41 (0.14)^{c}
C18:1n-9	10.3 (0.6)^{c}	13.4 (0.4)^{b}	14.6 (0.5)^{a}
C18:1n-7	0.48 (0.03)^{c}	0.80 (0.02)^{b}	0.94 (0.03)^{a}
C18:2n-6	26.5 (0.9)^{c}	43.3 (0.6)^{b}	48.6 (1.4)^{a}
C18:3n-6	-^{c}	1.001 (0.005)^{b}	1.325 (0.013)^{a}
C20:0	0.338 (0.005)^{c}	0.494 (0.002)^{b}	0.537 (0.07)^{a}
C18:3n-3	1.17 (0.06)^{c}	7.38 (0.18)^{b}	9.32 (0.07)^{a}
C20:1n-9	0.077 (0.013)^{c}	0.220 (0.012)^{b}	0.274 (0.004)^{a}
C18:4n-3	-^{c}	0.199 (0.018)^{b}	0.262 (0.013)^{a}
C21:0	0.042 (0.008)^{a}	0.018 (0.002)^{b}	0.015 (0.002)^{b}
C20:2n-6	-^{c}	0.038 (0.002)^{b}	0.045 (0.004)^{a}
C22:0	0.034 (0.012)^{c}	0.105 (0.002)^{b}	0.134 (0.013)^{a}
C22:1n-9	0.029 (0.003)^{a}	0.025 (0.003)^{a}	0.016 (0.002)^{b}
C23:0	0.0060 (0.0012)^{b}	0.014 (0.003)^{a}	0.008 (0.002)^{a}
C24:0	0.0110 (0.0006)^{c}	0.024 (0.007)^{b}	0.037 (0.005)^{a}

The same letter in superscript within the row indicates homogeneous groups established by ANOVA ($p < 0.05$). Nomenclature of FAs: Number after 'C' indicates the number of carbons, number after ':' indicates the number of double bonds, number after 'n' indicates the position of the first double bond from the methyl end of the FA.
